# Developing a Management Guide (the DemPower App) for Couples Where One Partner Has Dementia: Nonrandomized Feasibility Study

**DOI:** 10.2196/16824

**Published:** 2021-11-16

**Authors:** Reena Lasrado, Therese Bielsten, Mark Hann, James Schumm, Siobhan Theresa Reilly, Linda Davies, Caroline Swarbrick, Robyn Dowlen, John Keady, Ingrid Hellström

**Affiliations:** 1 Social Care & Society The University of Manchester Manchester United Kingdom; 2 Institute of Gerontology School of Health and Welfare Jönköping University Jönköping Sweden; 3 Division of Population Health The University of Manchester Manchester United Kingdom; 4 Faculty of Health Studies University of Bradford Bradford United Kingdom; 5 Faculty of Health and Medicine University of Lancaster Lancaster United Kingdom; 6 Centre for Cultural Value, School of Performance and Cultural Industries University of Leeds Leeds United Kingdom; 7 Division of Nursing, Midwifery and Social Work The University of Manchester Manchester United Kingdom; 8 Department of Health Care Sciences & Palliative Research Centre Ersta Sköndal Bräcke University College Stockholm Sweden

**Keywords:** dementia guide, self-management for couples with dementia, dementia self-help, dementia app, dementia resource, feasibility study, nonrandomized study, dementia intervention

## Abstract

**Background:**

Promoting the health and well-being of couples where one partner has dementia is an overlooked area of care practice. Most postdiagnostic services currently lack a couple-centered approach and have a limited focus on the couple relationship. To help address this situation, we developed a tablet-based self-management guide (DemPower) focused on helping couples enhance their well-being and relationship quality.

**Objective:**

The aim of this study is to investigate the feasibility and acceptability of the DemPower app.

**Methods:**

A nonrandomized feasibility design was used to evaluate the DemPower intervention over 3 months among couples where a partner had a diagnosis of dementia. The study recruited 25 couples in the United Kingdom and 19 couples in Sweden. Outcome measures were obtained at baseline and postintervention. The study process and interventions were evaluated at various stages.

**Results:**

The study was completed by 48% (21/44) of couples where one partner had dementia, of whom 86% (18/21) of couples accessed all parts of the DemPower app. Each couple spent an average of 8 hours (SD 3.35 hours) using the app during the study period. In total, 90% (19/21) of couples reported that all sections of DemPower were useful in addressing various aspects of daily life and helped to focus on how they interacted in their relationship. Of the 4 core subjects on which the DemPower app was structured, home and neighborhood received the highest number of visits. Couples used activity sections more often than the core subject pages. The perception of DemPower’s utility varied with each couple’s lived experience of dementia, geographic location, relationship dynamics, and opportunities for social interaction. A 5.2-point increase in the dementia quality of life score for people with dementia and a marginal increase in the Mutuality scale (+1.23 points) for caregiver spouses were found. Design and navigational challenges were reported in the DemPower app.

**Conclusions:**

The findings suggest that the DemPower app is a useful resource for couples where one partner has dementia and that the implementation of the app requires the support of memory clinics to reach couples at early diagnosis.

**Trial Registration:**

ISRCTN Registry ISRCTN10122979; http://www.isrctn.com/ISRCTN10122979

## Introduction

### Background

The progressive nature of dementia, with its symptoms of cognitive decline, poses challenges to relationships. Couples where one partner has dementia adapt to the transition from an interdependent relationship toward a relationship of caregiver-care receiver roles [1-4]. This transition can negatively affect a couple’s relationship, where the couple relationship is secondary to the care relationship. When the sense of couplehood is reduced, the risks of cognitive and functional decline increase [5] alongside the psychosocial dissatisfaction of both partners [6] and the need for special accommodations [7,8]. Extensive research has shown that the sense of couplehood is a crucial factor for well-being in everyday life among couples where one partner is diagnosed with dementia and for the prevention of negative consequences [9-11]. However, there is currently a gap in knowledge about how to support couples’ relationships and everyday lives in their own homes.

### eHealth and Self-management in Dementia

Interventions in dementia are often problem-based and target cognitive function, strain, and burden [1,2,12,13], and there is limited evidence of resource-oriented approaches. Self-management is a common feature in the treatment of chronic conditions. An increasing number of self-management eHealth services that consist of websites, applications, and monitoring are available for chronic conditions such as diabetes, chronic obstructive pulmonary disease, and heart failure [14]. There are also a small number of eHealth resources for informal caregivers of people with dementia [15]. The generic approach to self-management is often based on people’s perceived problems of a condition and deals with the management of symptoms [16]. This differs from the self-management approach that can be applied to dementia, where the focus is on managing challenges in everyday life from the perspective of quality of life, the abilities of people with dementia, and couples where one partner has dementia, and not solely on the condition and symptoms [17,18]. Bearing in mind this gap in positive, resource-oriented interventions for people with dementia and their partners, a couple self-management guide in the form of an app named DemPower was developed. The development of the guide was underpinned by salutogenic, resource-oriented, and strength-based approaches. The theoretical underpinning is discussed in detail in the protocol and DemPower development studies [17,19].

This study titled Living Life and Doing Things Together—work program 6 is part of the 5-year Economic Social Research Council and the National Institute for Health Research Neighborhoods and Dementia study (2014-2019) [20]. The study was funded in the United Kingdom under action point 12 of the first Prime Minister’s Challenge on Dementia [21] and was based in Manchester (United Kingdom) and Sweden.

A user-centered participatory design [22,23] guided the development of the DemPower app in the following 3 phases. Phase 1 involved a comprehensive literature review of couplehood and well-being in dementia, which informed a draft framework of themes identified as potential targets for the self-management guide [1,2]. Phase 2 explored the draft framework with 5 couples in Sweden, where a partner had a diagnosis of dementia. In this phase, the predetermined themes were presented to the couples to confirm or reject their relevance. Phase 3 authenticated the findings within expert groups of people with dementia and caregivers in Sweden and the United Kingdom. This phase enabled testing the empirical validity of the themes as sensitizing concepts, the transferability of findings to a UK context, and conversion into an app (for more information on the development phase, refer to the studies by Bielsten et al [17] and Lasrado et al [19]).

### Aims and Objectives

The overall aim of this study is to investigate the feasibility and acceptability of the DemPower app among couples living together at home, where one partner had dementia. The key objectives are to (1) evaluate the usability and acceptability of DemPower, (2) determine recruitment and completion rates, and (3) assess the suitability of the outcome measures for calculating the sample size of a full randomized controlled trial (RCT).

## Methods

### The DemPower App

**Figure 1:**
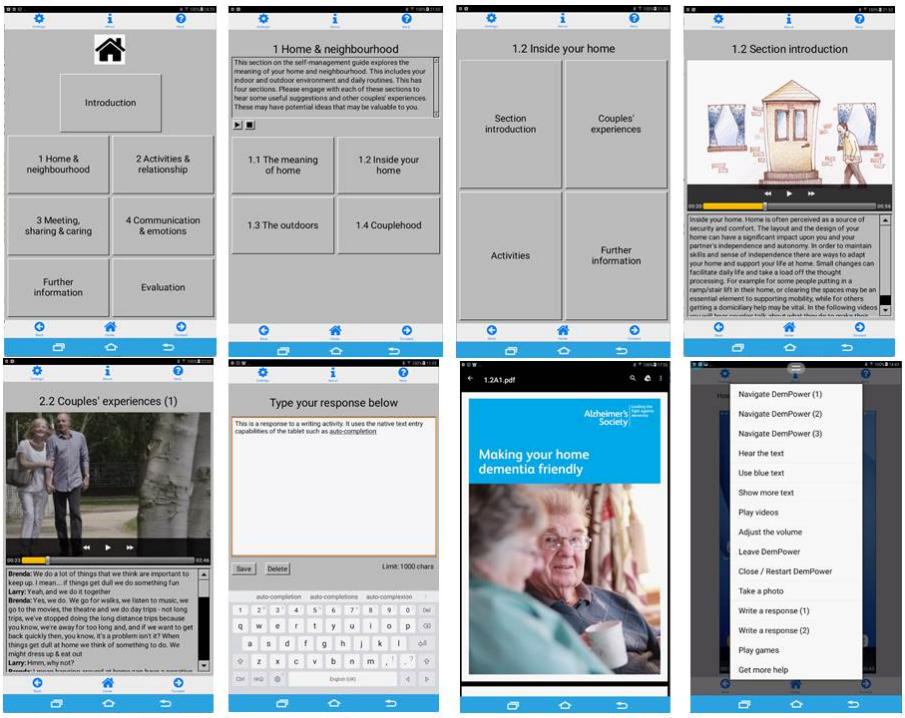
DemPower screenshots.

The DemPower app is a self-management resource guide intended for couples where one partner has a dementia diagnosis, and they live together at home. The app is structured around 4 themes with corresponding sections and suggestions for activities under each section (Table 1). The contents are storyboarded and converted into animated videos and films of couples who share their approaches to everyday life and situations. The home page of the app lists the core themes, navigational buttons are available at the bottom of the screen, and a help menu is available at the top of each screen throughout the app. Screenshots are shown in Figure 1. DemPower is a multimedia app with text, audio, and video sources. The app design focuses on making the interface simple and easy to access. User-centered and participatory approaches [24,25] informed the overall app design and concept.

The DemPower app focuses on enhancing couple relationships and managing everyday life. The couple participants were encouraged to complete all 4 themes or those parts they found relevant to their situation. The app guides the participants through introductory animated videos that describe the contents of each section, followed by videos of couples sharing their experiences. The aim of these videos was to provide participating couples with opportunities for reflection and active participation in the process by engaging in suggested activities. It takes between 10 minutes and 20 minutes to complete a section depending on the nature of the activities (Table 1). The app was installed on Samsung tablets, which were given to participating couples and which they could retain on completion of the study. The couples were encouraged to complete all parts of the app within the 3-month intervention period.

Participants were encouraged to contact the researchers (RL, TB, and RD) if they needed support and when they had completed all or the chosen sections under each theme. The researchers (RL, TB, and RD) were tasked with contacting participants every month by phone or email to ensure continued participation and to follow up on their progress. We also encouraged participants to make appointments if additional training or home visits were needed to address any challenges.

**Table 1:** DemPower content.

Themes			Activities
**1. Home and neighborhood**			
	1.1. The meaning of home	Take pictures	
	1.2. Inside	Use checklist to identify required changes or use SCIE app	
	1.3. Outside	Walk together, take pictures, and discuss	
	1.4. Couplehood	Describe positive relationship experiences, listen to music, and express emotions	
**2. Meaningful activities and relationships**			
	2.1. Physical exercise	Watch video, exercise, and keep a log	
	2.2. Doing things together at home and outside	List tasks to do together, choose one and engage	
	2.3. Individual activities	List individual activities and schedule time	
	2.4. Adapting activity to capability	Revisit the task list and discuss how to adapt	
	2.5. Mental exercise	Games	
**3. Meeting, sharing, and caring in your neighborhood**			
	3.1. Socializing with friends and family	Schedule meeting appointments, keep visitor log, and share communication sheet with family and friends	
	3.2. Meeting others who live with dementia	Visit social groups or dementia cafés	
	3.3. Informing each other and others	Share your experience with neighbors and discuss your experience	
**4. Managing communication and emotions**			
	4.1. Being a comfort and a friend	Discuss your approaches to comforting each other	
	4.2. Living as usual and keeping the routine	Plan a routine and display the routine	
	4.3. Stress	Listen to stress management audio and follow instructions	
	4.4. Conflicts	List strategies helpful for conflict management	
	4.5. Future and planning	Use the future planning checklist	
	4.6 Communication	Examine the listed strategies and add to it	

### The Study Design

A prospective, nonrandomized feasibility design was used to facilitate the assessment of study processes and to explore the usability and acceptability of the DemPower intervention. The study was approved by the National Health Service Research Ethics Committee (17/NW/0431) in the United Kingdom and the Regional Ethical Review Board in Sweden (Dnr: 2017 2017/281-31). The study was registered under the International Standard Randomized Controlled Trial registry (ISRCTN10122979).

### Setting and Participants

This was a multisite study based in North West England, the United Kingdom, and Linköping and Norrköping in Sweden. The participants in the United Kingdom were recruited via the Join Dementia Research (JDR) network at dementia cafés and through advertisements over a period of 12 months. The staff at these organizations disseminated the study information and obtained the initial expressions of interest. A researcher (RL) presented the study to groups at dementia cafés, and potential participants who learned about the study through posters contacted the researcher (RL) directly. In Sweden, memory clinics were the primary source of recruitment, and nurses approached potential participants at clinic appointments over a period of 12 months. A researcher (TB) then followed up with the potential participants over a further 6-month period, and recruitment in Sweden took 18 months. The researchers followed the process consent procedure [26] in both countries and obtained informed consent.

The detailed inclusion and exclusion criteria for recruitment are listed in Textbox 1 [19]. The participant characteristics were not limited to types of dementia, comorbidities, sexual orientation, age, profession, or social, cultural, or religious beliefs.

**Inclusion and exclusion criteria.**

**Inclusion criteria**

- Couples in which a partner or spouse has a diagnosis of dementia in the early to moderate stages. The stage will be identified either by a clinical team during referral or through self-report‎.
- The couples live together in their own homes (not residential care).‎
- Both partners understand and speak English (in the United Kingdom) or Swedish (in Sweden)‎.
- Couples have lived in a long-term relationship for 2 or more years.‎

**Exclusion criteria**

- Couples in which one or both partners are blind and might find it difficult to interact with DemPower.
- Any partner who has become completely immobile or bedbound and may not be able to engage with suggested activities‎.
- Both partners have a diagnosis of dementia. ‎
- Both partners in a couple in which one or both lack capacity or may have fluctuating capacity.

### Primary Outcome Measures

The primary outcome measure was intended to evaluate the usability and acceptability of DemPower and assess recruitment capability, sample size, and completion rates to determine whether a fully integrated clinical and economic RCT could be conducted.

#### DemPower Feasibility

The acceptability and suitability of DemPower was explored during the study and at the end of the study using a set of questionnaires adapted from Bowen et al [27], Craig et al [28], and Judge et al [29]. The System Usability Scale questionnaire on a 5-point Likert scale [30] was used to obtain participants’ perceptions of usefulness. Usage data were gathered from tablets at the end of the study. The app recorded a screen identifier (Multimedia Appendix 1) and timestamp every time the user moved to a new screen. Other measures can be deduced from the raw data.

#### Recruitment Capability

This study was informed by the recommendation of Aron et al [31] for assessing critical parameters such as recruitment and retention rate. Researchers (RL, TB, and RD) maintained a detailed record of the total number of target population accessed, recruited, and retained. Additional notes were maintained on the role of local organizations and colleagues, the time taken for recruitment, the number of contacts, visits, the challenges encountered, reasons for withdrawal from the study, and factors that influenced recruitment and study completion rates.

### Secondary Outcome Measures

We aimed to explore the acceptability and relevance of the secondary outcome measures used in the study to inform the selection of outcome measures in a full RCT to assess the effectiveness of the intervention. The outcomes of quality of life, self-efficacy, interconnectedness, and mutuality were measured using validated tools for both partners at baseline and postintervention. All outcome measures used in this study are listed in Table 2 [19]. The tools ranged from 1 to 15 items, with 3- to 5-point Likert scales and response options. Participants who chose to engage with only parts of the app completed postintervention outcome measures and end-of-study evaluation at a point when they felt they had finished the app. Where support was required, researchers (RL, TB, and RD) explained the questions and filled in the forms if participants were struggling to write or mark their responses using a pen and paper. Participants also commented on the ease of use of these tools.

**Table 2:** Outcome measures.

Outcomes	Tools	Description		Answered by
Quality of life	Quality of life in Alzheimer’s disease [32]		13-item tool Addresses mood, cognitive and functional ability, activities of daily life, and quality of relationships with family and friends A 4-point Likert scale ranging from “poor” (1p^a^) to “excellent” (4p) with a maximum score of 52	Both spouses or partners individually
Caregiver-related quality of life	Carer Quality of life [33]		A 7-item tool Addresses 5 negative and 2 positive dimensions of providing informal care A 3-point Likert scale from “a lot” (0p) to “no” (2p) for the negative dimensions and reversed scale for positive dimensions. The higher the score, the better the care situation.	Partner or spouse caregiver
Self-efficacy	General self-efficacy scale [34]		A 10-item tool Assesses coping skills and adaptation to situations Has a 4-choice response ranging from “not at all true” (1p) to “exactly true” (4p); Scores are summarized to a total score, and a higher score indicates a higher sense of self-efficacy.	Both spouses or partners individually
Interconnectedness	The Inclusion of Other in Self Scale [31]		A single item pictorial measure of closeness Assesses people’s sense of being interconnected to each other	Both spouses or partners individually
Mutuality	Mutuality Scale [35]		A 15-item Mutuality Scale Includes 4 dimensions—love and affection, shared values, reciprocity, and shared pleasurable activities Rated on a 4-point Likert scale between 0 “not at all” to 4 “a great deal”	Both spouses or partners individually
Health and social care service use	Service use questionnaire		The service use questionnaire was adapted from current service use questionnaires held by the investigators. It is to be refined after consultation with the study service user group. Covers key health and social care services Assesses the range of services used and the frequency of use The measure is to be administered by the researcher at baseline and at the end of follow-up assessments.	Both spouses or partners individually
Health status	5-level EuroQoL-5 dimension version [36]		Has a 5-dimensional structure (mobility, self-care, usual activities, pain or discomfort, and anxiety or depression) Each dimension has 5 levels: no problems, slight problems, moderate problems, severe problems, and extreme problems Allows estimation of quality-adjusted life years	Both spouses or partners individually
Quality of life	Dementia quality of life [37]		A condition-specific measure of health-related quality of life for people with dementia A 28-item tool Can be completed with the person with dementia or a main caregiver The measures cover 5 domains: daily activities and looking after yourself, health and well-being, cognitive functioning, social relationships, and self-concept Preference weights are available to allow estimation of quality-adjusted life years	Partner or spouse with dementia

^a^Scoring instructions for QOL-AD: points are assigned to each item as follows—poor=1, fair=2, good=3, excellent=4. The total score is the sum of all 13 items.

### Process Evaluation

The process evaluation was informed by the Medical Research Council’s guidance on complex interventions [28] and questions specific to the feasibility designs discussed by Bowen et al [27] and Orsmond and Cohn [38]. The relevance and significance of the DemPower intervention, its contents, design, and user interface were explored by the participants during the course of the study and at the end of the study using a questionnaire, usage data, and issue logs. This questionnaire included both close-ended and open-ended questions (refer to the protocol study by Lasrado et al [19] for the questionnaire) and was administered via an interview at home visits. The assessment of study procedures, recruitment and resource capability, and the relevance and feasibility of outcome measures were explored through a detailed analysis of researchers’ field notes and the end-of-study evaluation questionnaire presented to the participants.

### Data Management and Analysis

Data were analyzed using Stata software (version 14; StataCorp), and descriptive statistics were reported, such as measures of central tendency (mean and median) and spread (SD, IQR, and range). Responses to open-ended questions were processed using NVivo (version 11; QSR International) and analyzed thematically using the deductive approach. The outcome data were analyzed to determine whether there was sufficient change and variation in the measures, and these were checked for floor and ceiling effects. Recruitment and attrition rates were analyzed to assess the recruitment capability.

## Results

### Recruitment and Participant Characteristics

**Figure 2:**
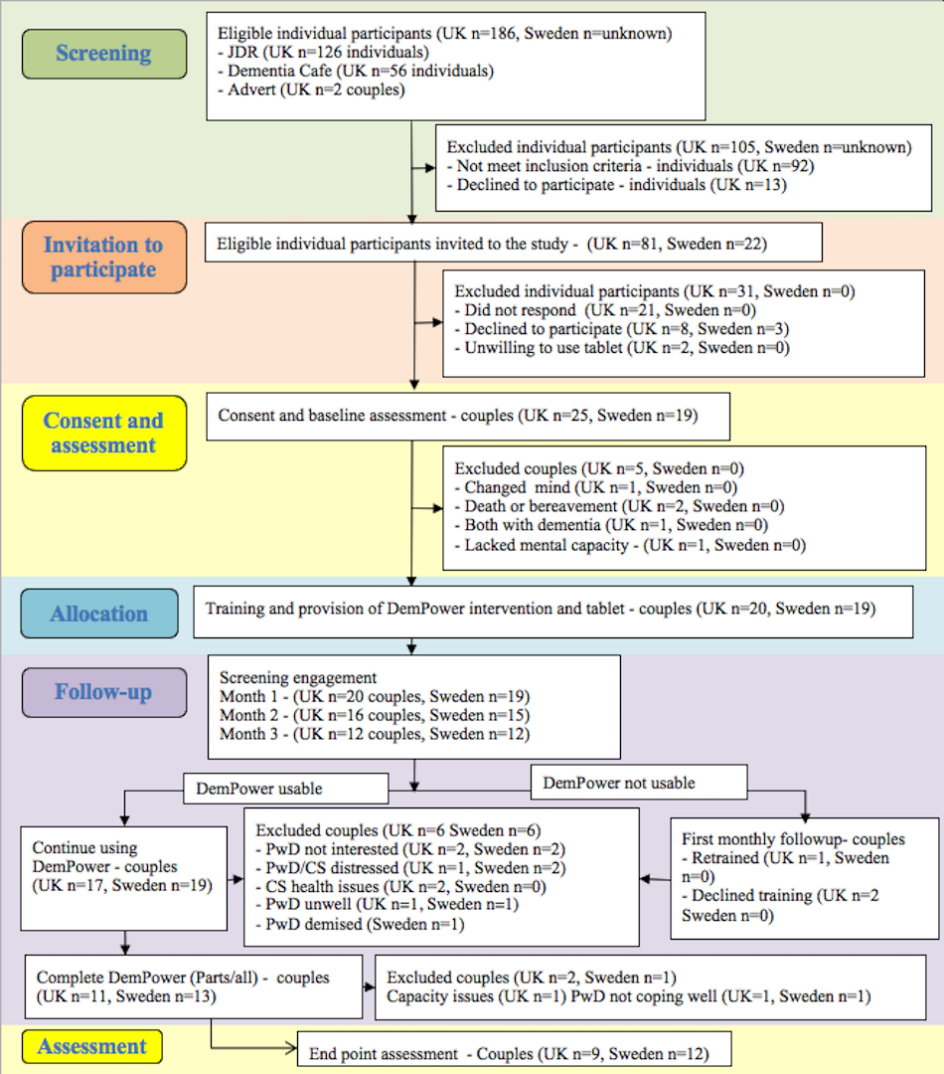
CONSORT (Consolidated Standards of Reporting Trials) flow diagram of participants. The number of people screened in Sweden was unavailable because of a lack of data from memory clinics. CS: caregiver spouse; JDR: Join Dementia Research; PwD: person living with dementia; UK: United Kingdom.

A total of 44 couples (United Kingdom, n=25; Sweden, n=19) were recruited at both sites between October 2017 and November 2018. The overall study completion rate was 48% (95% CI 33%-63%; United Kingdom: 9/25, 36%; Sweden: 12/19, 63%). Figure 2 outlines participant flow through the various stages of the study.

In the United Kingdom, 43.5% (81/186) of people with dementia and their caregiver spouses met the eligibility criteria and were identified via JDR, dementia cafés, and advertisements. A total of 50 (25 couples) participants consented to participate, representing a consent rate of 27% (95% CI 21%-34%). A total of 5 couples withdrew consent before the intervention, 6 during the study, and an additional 2 at follow-up. The recorded reasons for attrition were bereavement, declining mental capacity, both partners had dementia, challenging use of technology, lack of motivation, and ill health. In Sweden (Linköping and Norrköping), memory clinic nurses identified potential participants, and 44 met the eligibility criteria and 38 consented to participate. The total number of people screened for the study by the memory clinic nurses is unknown, as many nurses were involved, and records were not maintained. A total of 12 couples completed the intervention and the end of the study assessments. The reasons for attrition were disinterest among people with dementia, being unwell, and coming to terms with a recent diagnosis and one of the caregiver spouses wished to withdraw after they had viewed parts of the videos that discussed advanced stages of dementia, which they found distressing.

The demographic data from both sites revealed that 68% (13/19) of the participants with dementia in Sweden were over 71 years of age in comparison with 52% (13/25) in the United Kingdom. Swedish couples were potentially in a relationship for a longer duration than couples in the United Kingdom. A greater proportion of the participants from Sweden had a graduate education. The difference in education indicates potential socioeconomic differences in both countries. The gender differences in the study among people with dementia and caregiver spouses were more equal in the United Kingdom. In Sweden, 68% (13/19) of participants with dementia were men. It is also interesting to note that 36% (9/25) of participants with dementia in the United Kingdom had a mixed diagnosis, and another 36% (9/25) had Alzheimer disease. In Sweden, no participants had a mixed diagnosis; most (11/19, 58%) had Alzheimer disease and a more recent diagnosis (14/19, 74%; <2 years of diagnosis). In Sweden, people with more subtle or complex symptoms are referred to memory clinics and more likely to receive follow-up care, as primary care is limited in resources and competences [39]. Detailed demographics for both sites are presented in Table 3.

**Table 3:** Demographic characteristics of participants enrolled in the study^a^.

Characteristics			Persons with dementia, n (%)				Spouses (caregivers), n (%)		
			United Kingdom (n=24)		Sweden (n=19)		United Kingdom (n=24)		Sweden (n=19)
**Age (years)**									
	51-60	3 (13)		1 (5)		2 (8)		1 (5)	
	61-70	8 (33)		5 (26)		11 (46)		7 (37)	
	71-80	11 (46)		7 (37)		10 (42)		10 (53)	
	81-90	2 (8)		6 (32)		1 (4)		1 (5)	
**Gender**									
	Male	13 (54)		13 (68)		11 (46)		6 (32)	
	Female	11 (46)		6 (32)		13 (54)		13 (68)	
**Education**									
	Secondary	12 (50)		3 (16)		10 (42)		3 (16)	
	Advanced or upper secondary	4 (17)		4 (21)		3 (13)		5 (26)	
	Graduate	6 (25)		10 (53)		9 (38)		9 (47)	
	Postgraduate	2 (8)		2 (11)		2 (8)		2 (11)	
**Employment status**									
	Employed	4 (17)		0 (0)		8 (33)		3 (16)	
	Retired	20 (83)		19 (100)		16 (67)		16 (84)	
**Length of relationship (years)**									
	11-20	1 (4)		1 (5)		1 (4)		1 (6)	
	21-30	5 (21)		1 (5)		5 (21)		0 (0)	
	31-40	4 (17)		5 (26)		4 (17)		5 (28)	
	41-50	11 (46)		5 (26)		11 (46)		5 (28)	
	51-60	3 (13)		6 (32)		3 (13)		6 (33)	
	61-70	0 (0)		1 (5)		0 (0)		1 (6)	
**Type of diagnosis**									
	Alzheimer disease	9 (38)		11 (58)		N/A^b^		N/A	
	Frontal temporal	0 (0)		1 (5)		N/A		N/A	
	Lewy body	0 (0)		2 (11)		N/A		N/A	
	Mild cognitive impairment	2 (8)		0 (0)		N/A		N/A	
	Parkinson disease	0 (0)		1 (5)		N/A		N/A	
	Vascular	3 (13)		0 (0)		N/A		N/A	
	Mixed	9 (38)		0 (0)		N/A		N/A	
	Unspecified	1 (4)		4 (21)		N/A		N/A	
**Years since diagnosis**									
	<1	2 (8)		8 (42)		N/A		N/A	
	1-2	4 (17)		6 (32)		N/A		N/A	
	2-3	7 (29)		4 (21)		N/A		N/A	
	3-5	10 (42)		1 (5)		N/A		N/A	
	>5	1 (4)		0 (0)		N/A		N/A	

^a^Variable-specific column percentages do not always sum to 100 because of rounding. One UK couple did not provide demographic information. One Swedish caregiver did not provide information on relationship length.

^b^N/A: not applicable.

### DemPower Usability

Of the 21 couples who completed the study, only 86% (18/21) had accessed all sections of DemPower, and the average usage per couple was 8 hours (SD 3.35 hours) during the 3-month study period. Of the 4 themes, home and neighborhood averaged 250 visits; activities and relationships averaged 174 visits; meeting, sharing, and caring averaged 160 visits; and communication and emotions averaged 122 visits. The sections on home and neighborhood were in the first part of the app, which might explain the greater number of visits (Multimedia Appendix 2). However, the participant feedback detailed below highlights the role of this section in facilitating discussion and strategies for everyday life and associated challenges. Over the course of the study, participants visited suggested activity pages more frequently (mean 95) than the core content pages that had section-specific introductory videos (mean 71) or videos of couples (mean 69), for example, couples taking pictures, doing physical exercise, talking to each other, walking, and listening to music, and there was a home adaptation checklist.

### DemPower Acceptability

The theme-specific and end-of-study evaluation revealed that 90% (19/21) of couples acknowledged that all sections of DemPower were useful in addressing various aspects of daily life (Multimedia Appendix 3; for the evaluation questionnaire, see the protocol paper [19]). However, 24% (Sweden: 4/12, 33%; Manchester: 1/9, 11%) stated that the sections on mental activity (3/21, 14%), physical activity (2/21, 10%), managing stress (2/21, 10%), adapting activity (2/21, 10%), and meeting others with dementia (2/21, 10%) were less useful. As reasons for this, 2 couples gave their involvement in activities and their own exercise regime, and others said that they had their own strategies for addressing stress and that the suggested activities were less suitable.

A detailed analysis of couples’ perspectives on the meaning and usefulness of various parts of the DemPower app revealed that most couples found the sections on the home useful (13/21, 62% stated a great deal; 7/21, 33% stated somewhat). These sections helped them explore what home means to them, the need for adaptation, and how to adapt their home to meet changing needs. Parts of DemPower helped most couples focus on what they could do rather than what they could not do (18/21, 86%) and to recognize the importance of continued living as usual (20/20, 100%; a Swedish couple did not answer questions on themes 2, 3, and 4), and the app helped couples focus on how they interacted in their relationship and become more aware of the way they addressed everyday tasks (19/21, 90%):

It has made me think more about why we are doing things and making changes. It is food for thought.

MC16

More than half (11/20, 55%) of the couples indicated that DemPower helped them to recognize the need to maintain a social life, and 85% (17/20) of couples reported feeling encouraged and happy about meeting people. However, 60% (Sweden: 9/11, 82%; United Kingdom: 3/9, 33%) felt that sharing their experiences of dementia was hurtful and considered the activity burdensome rather than helpful. Recognizing the importance of a planned routine (20/20, 100%) and instructions for managing everyday communication (18/19, 95%) were found to be useful by most couples. A number of participants felt that the app helped somewhat and a great deal to address conflict situations (17/20, 85%), to practice relaxation (18/20, 90%), and to think about financial and legal (15/20, 75%) and care needs (17/20, 85%).

In total, 19% (4/21) of couples found information about support devices (locators, ID phone, and sensor lights) and contacts to discuss support needs irrelevant. All 4 couples were within 2-3 years of their diagnosis. In addition, 32% (6/19) of couples indicated that the information on counseling services was not very helpful as they lacked clarity on referral pathways.

Reminiscing about memorable moments (21/21, 100%), listening to music (19/21, 90%), and meeting people with dementia (15/17, 88%) rated high as suggested activities. These were followed by taking photos (18/21, 86%), physical exercise (15/19, 79%), and communication strategies (18/19, 95%). A few couples (4/19, 21%) said that the suggested exercises did not provide options to match different strength levels, and some felt encouraged to take further steps to maintain physical fitness. Activities that encouraged couples to plan for the future were rated as somewhat useful, indicating that the couples preferred to focus on the present:

Exercises too simple, would be good to get to choose some harder ones.

SC13

Bought a gym-card.

SC20

A total of 33% (7/21) of couples in the United Kingdom who rated the app positively also said that the app would be more relevant to people with limited knowledge and access to resources, those who are isolated and do not attend support or social groups, and those who are at the initial stages of diagnosis. Some (United Kingdom, n=3; Sweden, n=1) couples in the early stages of dementia found the content relevant to advanced dementia somewhat distressing and said that the app seemed more relevant for people at later stages of dementia. Those at a more progressive stage said that it was challenging for people with dementia to feel encouraged and focused (United Kingdom, n=2; Sweden, n=1), and they would have made better use of the app if they had received it earlier. A total of 2 people with dementia (United Kingdom, n=1; Sweden, n=1) and a caregiver spouse (United Kingdom, n=1) who expressed feelings of distress were offered support, and the distress protocol was followed. The development of the protocol was informed by current research and best practice evidence [40].

### Design and User Interface

Most participants at both sites said that the layout and overall design were simple, easy to use, visual, and helpful and had comprehensive information. A total of 8 (42%) participants found that using the same couples to narrate the story in various parts of the app helped them follow the storyline and coping methods. Some participants found having the same structure in all the sections of DemPower useful and liked the idea of being able to use it as and when they wished. A total of 13 couples (62%; United Kingdom, n=8; Sweden, n=5) used the help manual (paper and video) from time to time to guide them through the app:

Since using the app, we have done things that we wouldn’t have done before.

MC15 and MC16, eg, exercises, music, and word-search game

Some of the limitations raised by the participants included navigation concerns, confusion around indexing, lack of colors, and pointers to indicate where they were in their last session. Caregiver spouses often reported taking a leading role in initiating app usage and navigation, whereas partners with dementia used the activity sections more and at times returned to watching videos. A person with dementia from Sweden, who withdrew from the study because of the spouse caregiver’s lack of interest, used the app in a group session at a day care center with the help of a facilitator. This highlights the joint commitment and interest required from both partners to achieve relationship-focused outcomes.

The utility scale data (Table 4) revealed that couples in the United Kingdom liked to use the app more frequently than their Swedish counterparts. However, Swedish couples found the app easier to use and were more confident when using it. Participants in Sweden contacted the researcher more frequently via SMS text messages, phone, and emails than participants in the United Kingdom. This could potentially influence the usability of apps. There were mixed responses to how quickly participants could learn to use the app at both sites.

**Table 4:** Utility scale.

Characteristics	Sweden (13 couples)				United Kingdom (9 couples)		
	Codes 1 and 2 (disagree), n	Code 3 (neutral), n	Codes 4 and 5 (agree), n	Codes 1 and 2 (disagree), n		Code 3 (neutral), n	Codes 4 and 5 (agree), n
Like to use system frequently	3	7	3	2		3	4
System unnecessarily complex	10	2	1	4		3	2
System easy to use	0	2	11	2		3	4
Technical support required	8	3	2	6		1	1
Well integrated system functions	2	4	7	1		3	4
Inconsistency in the system	10	2	1	4		1	1
Quickly learn to use the system	1	7	5	3		1	4
Very cumbersome system	12	1	0	6		0	1
Confident using the system	0	3	10	3		1	4
Needed to learn a lot before use	11	2	0	6		1	1

### Outcome Measures

A total of 43 couples completed baseline measures, and 21 completed most follow-up measures (19 carers completed the Alzheimer’s disease quality of life [ADQoL] measure). Mean, SD, and mean change scores between baseline and follow-up are reported in Tables 5 and 6.

A 5.2-point increase, on average, was observed in the dementia quality of life (DEMQoL; measurement of health-related quality of life for people with dementia) score for participants with dementia, indicating a clinically significant change [41], particularly in the domains of social relationships and emotional well-being. There was a small increase, on average, in the Mutuality scale (+1.23 points) for caregiver spouses but no change in any of the other outcome measures. A comparison of these results with evaluation data suggests that DemPower had a positive effect on the couple relationship in terms of how they felt, expressed themselves, listened to others’ experiences, and used some of the suggested strategies. During the evaluation, participants said that it was helpful to have the flexibility to choose sections relevant to their situation and that using the app while on vacation or when having a dull moment was helpful to focus on their relationship and the practicalities of everyday life.

The degree of change (ie, the mean relative to the SD/range) on thē ADQoL scale was equivalent to that on the DEMQoL scale. Otherwise, the degree of change is much smaller. Although the domains explored in ADQoL and DEMQoL are similar, DEMQoL considers more detailed items under the rubrics’ emotional well-being and social relationships. Some of the individual, postintervention differences in the secondary outcome measures for people with dementia were large; for example, a 23-point decrease on the Mutuality scale or a 37-point increase on the DEMQoL. Such differences are not the norm but, in a sample of this size, can unduly influence the mean. There was some evidence of a ceiling effect in response to the inclusion of other in the self (IOS) scale. This was unsurprising given the narrow range and sensitivity of its measures. There was weaker evidence of a ceiling effect on the Mutuality scale. There was weak evidence of ceiling effects for caregiver spouses on self-efficacy, IOS, and ADQoL scales. Some couples felt that the mutuality questionnaire was too personal, and a few others found the IOS and self-efficacy scales difficult to understand.

**Table 5:** Outcome measures of people with dementia.

Outcome measures		Baseline (n=43)	Baseline (who also completed follow-up; n=21)	Follow-up (n=21)	Change (follow-up-baseline; n=21)
**Mutuality** **score**					
	Values, mean (SD)	48.31 (10.83)	50.57 (9.63)	49.81 (10.27)	−0.76 (6.71)
	Values, median (IQR)	51.0 (43.9 to 57.0)	54 (44 to 58)	53 (45 to 58)	0 (−3 to 1)
	Values, range	3 to 60	26 to 60	26 to 60	−23 to 15
**Self-efficacy**					
	Values, mean (SD)	28.48 (6.41)	30.04 (6.75)	30.89 (5.90)	0.85 (3.25)
	Values, median (IQR)	30 (25 to 32)	30.0 (27.8 to 34.0)	31 (29 to 34)	1 (−1 to 3)
	Values, range	11 to 40	14 to 40	15 to 40	−4.8 to 7
**Inclusion of other in the self**					
	Values, mean (SD)	5.79 (1.61)	6.43 (0.93)	6.19 (1.21)	−0.24 (1.00)
	Values, median (IQR)	6 (5 to 7)	7 (6 to 7)	7 (5 to 7)	0 (0 to 0)
	Values, range	1 to 7	4 to 7	3 to 7	−2 to 1
**Alzheimer disease quality of life**					
	Values, mean (SD)	36.92 (7.16)	38.19 (7.38)	40.33 (6.16)	2.14 (4.87)
	Values, median (IQR)	37.0 (32.0 to 41.2)	38 (33 to 43)	42 (37 to 44)	2 (−1 to 5)
	Values, range	18 to 50	25 to 50	28 to 50	−8 to 12
**Dementia quality of life**					
	Values, mean (SD)	85.46 (15.98)	88.67 (16.84)	93.86 (11.09)	5.19 (11.77)
	Values, median (IQR)	88 (73 to 98)	93 (79 to 103)	97.0 (85.0 to 99.1)	2 (−2 to 11)
	Values, range	45 to 112	48 to 112	73 to 112	−12 to 37
Carer quality of life		N/A^a^	N/A	N/A	N/A

^a^N/A: not applicable.

**Table 6:** Outcome measures of caregiver spouse.

Outcome measures		Baseline (n=43)	Baseline (who also completed follow-up; n=21)	Follow-up (n=21)	Change (follow-up-baseline; n=20)
**Mutuality score**					
	Values, mean (SD)	41.61 (11.13)	42.42 (11.13)	43.66 (12.16)	1.23 (4.38)
	Values, median (IQR)	44 (35 to 48)	44 (37 to 48)	46 (41 to 53)	1 (−1 to 4)
	Values, range	16 to 59	19 to 59	18 to 59	−10 to 9
**Self-efficacy**					
	Values, mean (SD)	31.95 (3.75)	33.10 (3.94)	33.10 (3.13)	0.00 (3.02)
	Values, median (IQR)	32 (29 to 35)	33 (30 to 36)	33 (31 to 35)	−1 (−1 to 1)
	Values, range	26 to 40	27 to 40	28 to 39	−5 to 8
**Inclusion of other in the self**					
	Values, mean (SD)	5.53 (1.59)	5.81 (1.33)	5.86 (1.11)	0.05 (0.59)
	Values, median (IQR)	6 (5 to 7)	6 (5 to 7)	6 (5 to 7)	0 (0 to 0)
	Values, range	1 to 7	2 to 7	3 to 7	−1 to 1
**Alzheimer disease quality of life**					
	Values, mean (SD)	40.40 (5.12)	41.58 (4.63)	41.63 (5.85)	0.05 (3.81)
	Values, median (IQR)	41 (38 to 44)	41 (39 to 46)	42 (36 to 46)	−1 (−2 to 2)
	Values, range	27 to 49	34 to 49	30 to 51	−7 to 8
Dementia quality of life		N/A^a^	N/A	N/A	N/A
**Carer quality of life**					
	Values, mean (SD)	7.60 (3.31)	7.48 (3.92)	7.52 (3.46)	0.05 (1.72)
	Values, median (IQR)	7 (5 to 10)	6 (5 to 11)	7 (5 to 11)	0 (−1 to 2)
	Values, range	1 to 14	1 to 14	3 to 13	−4 to 3

^a^N/A: not applicable.

Most people with dementia and caregiver spouses were able to complete the outcome questionnaires without much assistance. Some couples said that it was easier to respond to “yes” or “no” type questions rather than having to rate on a specific scale, whereas some couples experienced difficulty in interpreting the IOS and found the Mutuality scale too personal. A few people with dementia and caregiver spouses had difficulty completing the self-efficacy questionnaire, and the researcher had to explain the questions. The spousal caregivers said that the carer quality of life questionnaire items were irrelevant, as most people with dementia were able to execute daily tasks independently.

## Discussion

### Principal Findings

The feasibility and acceptability of the DemPower app was explored at various stages of the study by investigating participants’ opinions of the content, design, and delivery. The DemPower app is a self-management guide intended to support both persons with dementia and their partners in their efforts to enhance well-being and relationship quality. The results show that the topic areas addressed in the app were meaningful and relevant to everyday life situations, although their utility varied with couples’ trajectory through dementia and their general well-being. Evidence confirms that recognizing a person with dementia and the family caregiver’s position in their aging trajectory is essential in understanding how people make use of the support and perceive its effectiveness [42-44]. The videos of couples sharing their experiences and the active prompts in DemPower were reported to have encouraged couples to reflect on their own approaches to everyday activities, discuss their relationship, recognize both positive and challenging aspects of their life together in the context of dementia, and share experiences with each other. DemPower further challenged their own perceptions of dementia and their everyday choices. This might indicate the change observed in the DEMQoL scores for people with dementia and the marginal increase in the Mutuality scale for spouse caregivers. These results reveal that the self-management approach, concepts, videos, and suggested strategies for couples as a dyad are promising. This is consistent with the findings of a recent systematic review that found that a caregiver’s emotional withdrawal can negatively affect the behavior of a person with dementia [45], which reaffirms our approach of actively involving both partners in couple-focused self-management.

### Usability and Acceptability of DemPower

Examining the cultural adaptability of DemPower in both countries revealed that relationship dynamics, perception and acceptance of the condition, varied opportunities for social interaction, and geographic location informed the couples’ usage. For example, couples in the United Kingdom had better access to dementia cafés and activity-based groups such as reading, walking, choir, and art groups. Although not all couples welcomed the idea of attending groups, those who responded to the suggested activity said that their misconceptions were challenged and that they enjoyed the group, made friends, and continued attending the group activities. Couples had limited opportunities for socializing in groups in Sweden, and more so in rural locations in that country. Most Swedish couples moved between their summer and winter homes, which means that activities changed according to their location. However, the couples said that the videos on DemPower helped them learn about other couples’ experiences, mutual interactions, and to feel that they were not alone in the way they experienced the situation. Most couples said that they would consider using DemPower in the future.

The use of technology and availability of DemPower on a handheld device provided couples with easy access to resources and suggestions that were relevant to everyday life situations, regardless of their location. All couples in both countries said that they were comfortable using a tablet device; however, navigational challenges in the DemPower app and lack of motivation in a few people with dementia have been reported. Both partners engaged actively in individual and couple-focused activities and watched videos, regardless of design-related challenges. Increasing evidence suggests that technology-based interventions in dementia that encourage active involvement contribute to better quality of life and quality of relationships [46,47]. The parts of the app that discussed advanced dementia were reported as distressing for some couples, and these parts were likely to discourage these couples from using the app. However, some studies have emphasized the need to address the future to create a sense of normalcy and deal with fears [48]. Other research into sensitive topic areas has highlighted participant distress; however, no research has discussed any long-term impact or continued distress caused by research participation [49]. The core contents of DemPower were carefully considered, informed by current evidence, and in consultation with people with dementia and their partners [17,50]. However, the presentation and design of DemPower needed further consideration to facilitate participant preparedness and to allow participants to select topics that were relevant to the participants’ stage of dementia and at the time of their choosing.

### Recruitment and Completion Rates

The study obtained tremendous support in the recruitment of participants from organizations in both the United Kingdom and Sweden. The JDR network in the United Kingdom screened most of the potential participants there, but this organization was independent of the clinical care team. The Swedish memory clinic nurses approached potential participants during their clinic appointments. It is likely that the signposting of study by the care team might have influenced the recruitment and retention rates in Sweden and introduced selection bias to a certain extent. For example, the memory clinics in Sweden usually follow up with persons with dementia with more complex symptoms up to 6 months after diagnosis. The differences in the type of dementia, age, education, gender, and the length of time since the diagnosis in this study highlight the need to carefully consider these variables in the design of a future trial.

Planned strategies that address any unforeseen delays in intervention delivery, being mindful of motivational issues in both partners, promoting interest in the use of technology, and maintaining continuity in researcher-participant contact are some of the recommendations for a future trial. To detect a 4-point change in the DEMQoL, assuming an SD of 15 points, a correlation of 0.6 between baseline and follow-up scores, and an 80% retention rate at follow-up, 354 couples would need to be randomized for a definitive RCT to achieve 80% power (480 couples for 90% power) [41]. Memory clinics in Sweden and dementia advisers in primary care and third-sector organizations in the United Kingdom are most likely to be the point of delivery in the future.

### Suitability of the Outcome Measures

Outcome measures, such as quality of life, self-efficacy, and relationship-focused tools, could be considered to evaluate the changes and the impact the app has on the everyday lives of couples. It is important for these outcome measures to reflect what is important to people living with dementia [44] and consider the core outcome set for evaluating community-based interventions for people with dementia [51]. In view of the study results, measures of social well-being, relationship quality, positive feelings, and strength-based perspectives need particular focus in the future. The wider literature acknowledges the relevance of these domains and their potential to capture the experiences of people with dementia and family caregivers [51,52]. The measures also need to be mindful of the intrusive nature of the questions, especially those that assess relationship quality and emotional well-being, to determine whether self-administration or the interview method is ideal. Although the study found that it was feasible to use interviewing strategies to obtain responses to open-ended questions in the evaluation and administering outcome measures, the method limited the exploration of new themes arising from the responses. Hence, the use of both a questionnaire and in-depth interviews to assess outcomes and perform evaluations at various stages of the study is important for future consideration.

### Conclusions

The findings suggest that the DemPower app is a meaningful resource for addressing various aspects of daily life and interactions in couple relationships where one partner has dementia. However, whether DemPower is more relevant for people with a recent diagnosis of dementia needs to be explored. The design and organization of app contents must be revised before further implementation and testing of the app. A larger sample size, longer follow-up periods, and various control groups (including couple groups rather than individual couples) need to be considered to test the effectiveness of the app. Important outcomes for the couples in this study were to be able to continue as usual, focus on strengths, on social well-being, and mutual relationship quality. These factors need to be considered when identifying relevant outcome measures for future trials.

## Appendix

Multimedia Appendix 1 Usage log lookup.

Multimedia Appendix 2 Number of visits per screen.

Multimedia Appendix 3 Evaluation data summary.

## Abbreviations

ADQoL: Alzheimer’s disease quality of life
DEMQoL: dementia quality of life
IOS: inclusion of other in the self
JDR: Join Dementia Research
RCT: randomized controlled trial
