# Supplementary figures and images for: Developing a Management Guide (the DemPower App) for Couples Where One Partner Has Dementia: Nonrandomized Feasibility Study
Source: JMIR Aging. 2021 Nov 16;4(4):e16824. doi: 10.2196/16824 (PMC8663680; doi:10.2196/16824)

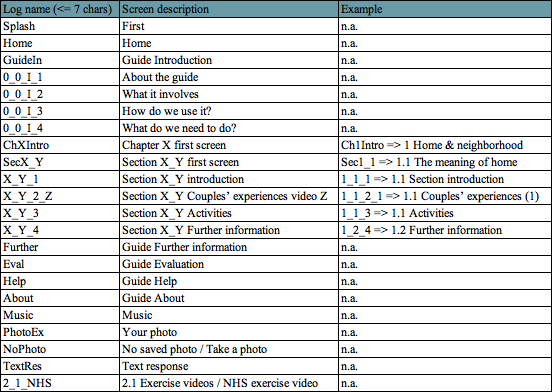

Supplement: Multimedia Appendix 1 [file aging_v4i4e16824_app1.png]

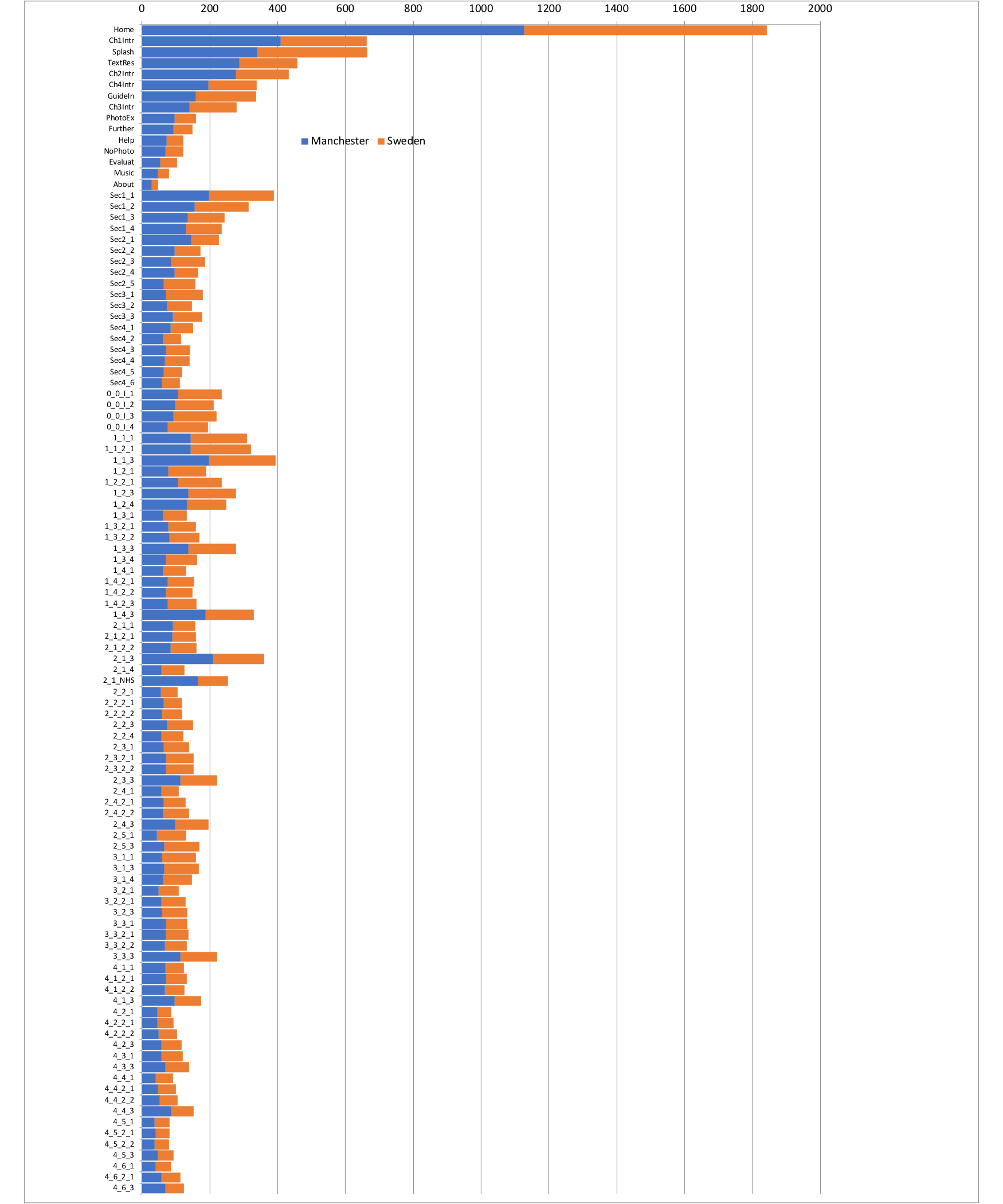

Supplement: Multimedia Appendix 2 [file aging_v4i4e16824_app2.png]
